# Diagnostic Delays Are Common, and Classic Presentations Are Rare in Spinal Epidural Abscess

**DOI:** 10.5811/westjem.24985

**Published:** 2025-02-24

**Authors:** Edward J. Durant, Sarabeth Copos, Bruce F. Folck, Meredith Anderson, Meena S. Ghiya, Erik R. Hofmann, Peter Vuong, Judy Shan, Mamata Kene

**Affiliations:** * Kaiser Permanente Bernard J. Tyson School of Medicine, Department of Clinical Sciences, Pasadena, California; † The Permanente Medical Group, Department of Emergency Medicine, Pleasanton, California; ‡ Kaiser Permanente Central Valley, Department of Emergency Medicine, Modesto, California; § Kaiser Permanente Northern California, Division of Research, Pleasanton, California; || University of California San Francisco School of Medicine, Department of Clinical Sciences, San Francisco, California

## Abstract

**Introduction:**

Spinal epidural abscess (SEA) is a rare surgical emergency of the spine that can result in permanent neurological injury if not diagnosed and treated in a timely manner. Because early presentation can appear similar to benign back or neck pain, delays in diagnosis may be relatively common. We sought an improved understanding of the characteristics associated with SEA and frequency of delays in SEA diagnosis.

**Methods:**

We conducted a retrospective cohort study of adult patients with new magnetic resonance imaging-confirmed SEA from January 1, 2016–December 31, 2019 in an integrated healthcare system. We applied electronic data abstraction and focused manual chart review to describe potentially SEA-related ambulatory and emergency visits in the 30 days prior to SEA diagnosis, and patient characteristics including comorbidities, potential risk factors, and presenting signs and symptoms. We described the frequency of potential delays in diagnosis and of previously described clinical characteristics and risk factors for SEA.

**Results:**

Spinal epidural abscess was diagnosed in 457 patients during the study period, 178 (39%) of whom were female, with median age 63 years (interquartile range 45–81 years). More than two-thirds of patients had at least one visit prior to diagnosis (323, 71%), and SEA location was most commonly the lumbar spine (235, 51%). Although over 90% of patients presented with back or neck pain or tenderness, the classic triad of back pain, fever, and neurologic symptoms was present in only 10% of patients. Diabetes mellitus and infection in the prior 90 days were common, while injection drug use, chronic steroid use, HIV infection, and solid organ transplant were rare.

**Conclusion:**

In an integrated healthcare system, 71% of patients with spinal epidural abscess had potentially related ambulatory care or emergency visits in the 30 days prior to diagnosis. Diagnosis of SEA remains challenging, with multiple visits common before the diagnosis is clear.

## INTRODUCTION

Spinal epidural abscess (SEA) is a rare condition with increasing incidence over the past decade, which if not promptly diagnosed and treated can lead to permanent and devastating neurologic disability.^1^,^2^ It is a condition associated with high morbidity resulting in permanent neurologic deficit in approximately one-third of SEA patients.^3^ Accurate diagnosis requires mobilization of magnetic resonance imaging (MRI), the diagnostic gold standard, but it is a relatively scarce and time-consuming resource in emergent situations. The clinical presentation of SEA can vary, however, and many patients have multiple visits before diagnosis.^4^ The most common complaint in SEA malpractice claims is delay in diagnosis, and mean claim payouts are between $4–5 million dollars.^5^,^6^

Despite our understanding of the diagnostic urgency of SEA, clinical guidance to determine which patients need emergent MRI is sparse. Beyond a high index of clinical suspicion, the classic triad of back pain, fever, and neurologic deficit is present in only a minority of patients with SEA.^1^ Obtaining an MRI in all ED patients with neck or back pain, however, is impractical and would lead to low-yield over-testing. This dilemma may give rise to delays in diagnosis, which are costly and associated with worse long-term neurologic outcomes.^4^,^6^–^9^

Previous literature on SEA is comprised largely of case series over long periods or single institutions, and published risk factors across varied study populations may include coincident conditions rather than causal risk factors, complicating risk assessment.^2^,^3^,^10^–^15^ Small studies and case series postulate characteristics associated with SEA, including advanced age, gender, diabetes, end-stage renal disease, history of spine surgery or dental work, immune compromised state, injection drug use, trauma, and alcohol use disorder.^2^,^8^,^16^,^17^ Laboratory and exam findings associated with SEA include elevated serum inflammatory markers (C-reactive protein [CRP], erythrocyte sedimentation rate [ESR], leukocytosis), fever, back pain, and progressive neurologic deficit.^14^,^18^ Given the relatively small case series and variation in described SEA characteristics, a larger dataset over a shorter time period would add to our understanding of the disease.

We sought to describe SEA characteristics and the frequency of potential delay in diagnosis in a large integrated healthcare system. We hypothesized that classic presentations would be rare and that potential delays in diagnosis would be common.

## METHODS

### Study Setting and Participants

We conducted a cross-sectional study of adult (≥18 years of age) patients diagnosed with SEA from January 1, 2016–December 31, 2019 at Kaiser Permanente Northern California (KPNC), an integrated healthcare system. KPNC provides care to over four million individuals who are representative of the surrounding community with respect to demographics, socioeconomics and medical conditions.^19^,^20^ Patients were included in the study if they had a new diagnosis of SEA (spine MRI with epidural fluid collection or phlegmon within three days of diagnosis documentation), and were health plan subscribers in 9 of the 12 months prior to diagnosis (to ensure complete health records for data extraction).

**Population Health Research Capsule**

What do we already know about this issue?

*Spinal epidural abscess (SEA) is a rare surgical emergency with variable clinical presentations that can result in permanent neurological injury if not promptly diagnosed.*

What was the research question?

*How common is delay in diagnosis, and what are the characteristics of patients with SEA?*

What was the major finding of the study?

*Most (70.7%) patients had a delay in diagnosis. Only 10.7% of patients had the triad of back or neck pain, fever, and weakness.*

How does this improve population health?

*This study highlights challenges associated with the timely diagnosis of SEA and the lack of any specific clinical or sociodemographic factors associated with this delay.*

### Study Design

The KPNC Institutional Review Board granted permission to publish fully anonymized, aggregate data, with a waiver of individual patient consent. We used electronically extracted and manually reviewed data to confirm SEA diagnosis and ascertain patient and SEA characteristics, as well as history of potentially related ambulatory or emergency department (ED) visits in the 30 days prior to diagnosis. Patient demographics, comorbidities, social history (injection drug use, illicit drug use, and alcohol use disorder), infection 90 days prior or spine instrumentation 30 days prior to diagnosis, and presence of indwelling vascular catheter were all evaluated.

#### Main Variable of Interest

Our main variable of interest was the prevalence of potential diagnostic delay, defined as a visit in the 30 days prior to SEA diagnosis in ambulatory care (primary care, spine surgery, physical medicine and rehabilitation, neurology, and orthopedics) or the ED with documentation of any of the following: back or neck pain; fever or chills; radicular pain; sensory changes; motor weakness; or cauda equina symptoms or findings.

#### Covariables

Electronic variables were extracted from the electronic health record (EHR) (Epic Systems Corporation, Verona, WI) and its associated databases by experienced data analysts (MA, BFF). The following data elements were extracted electronically: age; sex; ethnicity/race; English language preference; and neighborhood deprivation index (NDI, a composite measure of socioeconomic status); body mass index (BMI); solid organ transplant; alcohol use disorder; chronic steroid use; chronic liver disease; chronic kidney disease; diabetes; HIV infection; 90-day prior sepsis, bacteremia, endocarditis, or joint, urinary or skin/soft tissue infection (International Classification of Diseases, Rev 10 [ICD-10] codes).

#### Structured Electronic Health Record Review

Manual chart review was performed by investigators who were trained prior to data collection. A coding manual containing a priori definitions of positive findings (explicitly documented notation of presence) and negative findings (explicit documentation of negative findings or no documentation) was reviewed with all investigators prior to commencing chart review, and MVK trained reviewers on the data collection tool and process. Inclusion criteria, exclusion criteria, and variables for case selection were defined a priori. Although investigators were not blinded to the outcome, variables and their coding were pre-defined and discrepancies were adjudicated by the two co-principal investigators (SMC and MVK), with a consensus decision based on definitions in the coding manual.

All chart abstractors received standardized training on data collection methods and use of the electronic data collection instrument, which was modified to its final form after pilot testing. SMC and MVK answered and arbitrated all coding questions and monitored data collection activities by reviewing each abstractor’s performance at regular intervals throughout the abstraction period. Inter-rater reliability was tested as excellent (kappa = 0.8649, 95% confidence interval 0.7180–1.0000) between the co-principal investigators who performed the majority of chart reviews and supervised chart abstraction.

All matching ICD-10 code diagnoses during the study period were extracted from the KPNC EHR. Chart abstractors then manually validated the presence of a new SEA diagnosis by chart review. Missing values for demographic and clinical variables were not imputed and are enumerated in their respective tables. We limited our analyses to descriptive statistics and univariate regression; so, these missing variables were not critical. All criteria for health record review as outlined by Worster and Bledsoe were followed except for calculating IRR for the non-co-PI chart abstractors, as mentioned previously.^21^

Specific manually abstracted elements were confirmation of new SEA diagnosis and location (MRI findings of epidural fluid collection or phlegmon); social history (unhoused status, current use of tobacco, illicit drugs, injection drugs, and/or alcohol use disorder); previous 30 day spine instrumentation (epidural injection, paravertebral facet joint denervation [radiofrequency neurolysis]; paravertebral facet joint injections or blocks; sacroiliac joint injections; lumbar/pre-sacral spinal fusion surgery; spinal decompression surgery; artificial intervertebral disc replacement; release of spinal cord, excision of joint or disc, spinal cord stimulator); 90 day prior antibiotic prescription; and presence of indwelling vascular catheter (peripherally inserted central catheter, hemodialysis catheter, chemotherapy port). For the index visit (visit during which a spine MRI was ordered) and for visits in the 30 days prior to diagnosis in ambulatory care or the ED, we extracted presenting signs and symptoms consistent with SEA (i.e., fever or chills, documented temperature at or above 38°C or 100.4°F, complaint of back or neck pain, spinal tenderness to palpation, radicular pain, sensory changes, motor weakness, cauda equina constellation [urinary retention >200 milliliters, new urinary or stool incontinence, decreased rectal tone and/or saddle or perineal anesthesia]).

### Statistical Analysis

We calculated descriptive statistics (frequencies, proportions, means and medians) for demographic and clinical characteristics of patients diagnosed with SEA. Categorical variables, including risk factors, presence of sensory, motor, or perineal findings on exam at the time of index diagnosis, are presented as proportions. Continuous variables including laboratory tests (CBC, ESR, CRP), are presented as means with standard deviations or median values and interquartile ranges. We conducted a bivariate analysis to identify covariates associated with the potential delay in SEA diagnosis using chi-square or Fisher exact tests for categorical variables, and *t*-test or non-parametric tests for continuous variables. We conducted analyses using SAS 9.4, (SAS Institute In., Cary, NC) with the threshold of significance set at two-sided *P*<0.05.

## RESULTS

After applying exclusion criteria, the 457 patients included in the study cohort (Figure 1) , had a median age of 63 years (interquartile range 45–81 years), and 178 (39%) were female. Over two-thirds of patients (323, 70.7%) had at least one visit in the 30 days prior to SEA diagnosis for back or neck pain, fever, radicular pain, focal weakness, or numbness. Race/ethnicity and primary English language preference were similar between patients with 30-day prior visits and those without. Chronic steroid use, solid organ transplant, HIV infection, and injection drug use were infrequent, while diabetes mellitus and recent infection were common (Table 1). Of these variables, only chronic steroid use (9.0% vs 2.2%) and unhoused status (0.3% vs 3.7%) showed a statistically significant difference in delay vs no delay in diagnosis, although very few of our patients were chronic steroid users or unhoused (32 and 6, respectively).

At diagnosis, at least one element of the classic triad of back or neck pain, fever, and weakness was commonly present, with 91% of patients presenting with back or neck pain; however, only 10.7% of patients had all three triad elements (Table 2). Elevated temperature was present in 27% of patients with potential delay and 34% without potential delay. Weakness or motor findings were present in 27% and 31% of patients with and without potential delay, respectively. Although the difference in the proportion of weakness or motor findings between patients with and without potential delay did not reach statistical significance (27% vs 31%, *P*=0.09), the trend suggests a possible association (Table 2).

Among patients with a delay in diagnosis, most (69%) had two or fewer potentially related visits prior to diagnosis with a mean of 2.18 visits (95% CI 2.03–2.34). The number of visits prior to diagnosis in this group is tabulated in Table 3. Among this group, the average time between their initial visit and the index visit (diagnosis) was 12.82 days (95% CI 11.79–13.84).

## DISCUSSION

In this retrospective study of ED patients with SEA, 70.7% of patients had at least one ambulatory care or ED visit in the 30 days prior to diagnosis (visit documentation of back or neck pain, fever or chills, radicular pain, sensory changes, motor weakness, or cauda equina symptoms). Previously described risk factors of chronic steroid use, HIV infection, injection drug use, and solid organ transplant were infrequently observed, while diabetes mellitus was present in over one-third of the cohort. Fever and neck or back pain were common.

Our study highlights some of the challenges in diagnosing SEA and aligns with other literature on SEA characteristics. Not only was at least one prior potentially related visit common among patients diagnosed with SEA, but the most common presenting symptom—back or neck pain or tenderness—has a broad differential diagnosis with many benign etiologies. Our finding that fever and back or neck pain were common in both groups, those with and those without potential delay in diagnosis, underscores the challenge of finding serious causes of back or neck pain in ambulatory and emergency settings. A systematic review of 40 publications on SEA found the most common characteristics associated with SEA were fever and spinal pain.^22^ Proposed clinical risk prediction scores for SEA or pyogenic spinal infection include clinical and historical features such as injection drug use, liver disease, diabetes, spine instrumentation, recent infection, and indwelling catheters as well as progressive neurological deficit, CRP and fever, and may aid in identifying potential SEA among myriad other causes of back pain.^23^–^25^ Several of these characteristics were common in our cohort, including diabetes, fever, back or neck pain, and recent infection.

We did not observe racial or ethnic disparities in association with potential diagnostic delay nor was English language preference or socioeconomic status associated with potential delay in diagnosis in bivariate analyses. Larger studies report that Black and Hispanic non-White patients may be less likely to undergo advanced imaging in the ED, and racial disparities associated with delayed diagnosis of conditions from malignancy to infection are well described.^26^–^30^ The observation that age, race/ethnicity, and sex were not associated with potential diagnostic delay in our cohort may reflect more equitable access to health services among members of an integrated healthcare system; however, absent a large enough sample to perform multivariable analyses, interactions between these characteristics may be present, complicating the interpretation of these results. We may have inadvertently excluded patients with other risk profiles including injection drug use, unhoused status, or alcohol use disorders from our analysis as they may be under-represented within our system.

The timing of diagnosis and intervention remains of paramount importance in SEA. Although robust data on the optimal timing of surgical intervention is sparse, limited data describes more favorable neurologic outcomes with earlier (<24 hours) surgical management.^31^ Our observation of a non-statistically significant association (*P* = 0.09) between weakness or motor findings and delay in diagnosis aligns with the expectation that patients experiencing longer delays may manifest neurological motor weakness over time. Had we identified additional factors associated with delayed diagnosis, these could have served as potential red flags for clinicians to consider in patients presenting to the ED with back pain or fever. Back pain, however, is the eighth most common reason for ED presentation, and fever is the fifth.^32^ Obtaining advanced imaging on every patient with back pain, or even the combination of back pain and fever, is infeasible and unnecessary.

One of the challenges in diagnosing SEA is that presentations might not be classic until SEA has progressed substantially; so delays from the first symptom to definitive SEA diagnosis may be common. In fact, increased time to diagnosis has been noted among patients with errors in SEA diagnosis and frequency of diagnostic delay may be as high as 75%.^4^,^24^ Given the prevalent allegation of delayed diagnosis and high malpractice settlements in successful SEA claims, non-operative clinicians, including emergency physicians, may be at higher risk of litigation.^6^ A multitude of proposed risk factors and the frequency of back pain-related ED visits make diagnosing SEA akin to finding a needle in a haystack; one multistate study noted a 0.1% rate of intraspinal abscess among 1.3 million patients discharged from the ED with a non-specific back pain diagnosis.^33^ In our cohort, the prevalence of many frequently described SEA risk factors was similar among patients with and without prior visits (potential diagnostic delays), underscoring that SEA may persist as a difficult diagnosis to make without the “tincture of time” or repeated evaluation.

## LIMITATIONS

This retrospective study had several limitations. Only patients with health plan membership were included, but SEA risk factors encompass some variables that may be more common in patients without regular care access (eg, homelessness, substance use); thus, our ability to discern significant associations between these less common risk factors and diagnostic delay was limited. However, KPNC members are representative of the surrounding communities with respect to demographics, comorbidities, and health status. Because the study was designed to examine visits prior to diagnosis, there was no feasible way to include patients without health plan membership, who would not have had prior outpatient visits in our system. We relied on chart review for some variables as we could not feasibly extract these datapoints electronically. Chart review followed a priori definitions established by the PIs based on published definitions in the literature, with training of chart reviewers on the most complex chart-review variables. We relied on documentation of key risk factors (social history) that depend on the clinician asking the question and documenting the answer in their note.

Missing data were coded as absence of that variable if not documented or if documented as negative. Although we constructed an extensive list of variables previously studied, it is possible that confounding variables or unknown significant risk factors were not included. We included all SEA cases over a consecutive four-year period, but SEA is rare; so our sample was underpowered to perform adjusted analyses of characteristics associated with potential delay in diagnosis. Nevertheless, it is a larger sample in a shorter period than current studies.

## CONCLUSION

Our study highlights some of the challenges associated with the timely diagnosis of spinal epidural abscess. Potential delay in diagnosis appears to be the rule rather than the exception, and we did not find any specific clinical or sociodemographic factors that were associated with this delay. It is unclear whether this reflects the inherent challenge of diagnosing SEA or whether participation in an integrated healthcare system may attenuate some of these effects. Further studies could help uncover some of the reasons for delay, as well as better elucidate risk factors for SEA.

## Figures and Tables

**Figure:**
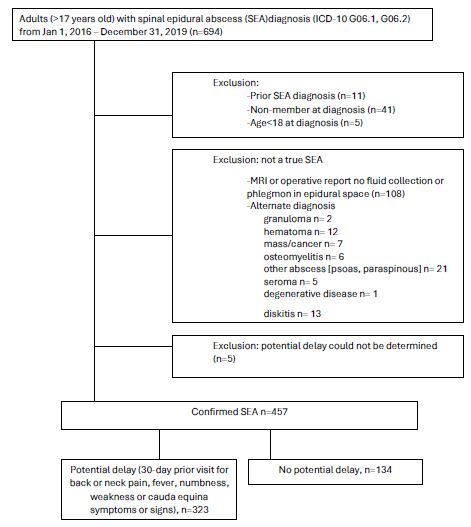
Cohort assembly of patients with spinal epidural abscess.

**Table 1:** Characteristics of patients diagnosed with spinal epidural abscess.

Characteristics*	All SEA patients (N=457, 100%)	Potential delay in diagnosis (n=323, 70.7%)	No potential delay in diagnosis (n=134, 29.3%)	*P*-value†
Age (Years), median (IQR)	63.0 (54.0–72.0)	63.0 (55.0–72.0)	63.0 (53.0–71.0)	0.90
Sex, n (%)				0.70
Female	178 (39.0)	124 (38.4)	54 (40.3)	
Male	279 (61.0)	199 (61.6)	80 (59.7)	
Race/Ethnicity, n (%)				0.87
White	273 (59.7)	191 (59.1)	82 (61.2)	
Asian/Pacific Islander	47 (10.3)	33 (10.2)	14 (10.4)	
Black	48 (10.5)	36 (11.2)	12 (9.0)	
Hispanic/Latinx	67 (14.7)	49 (15.2)	18 (13.4)	
Other‡	22 (4.8)	14 (4.3)	8 (6.0)	
Preferred language English§, n (%)	432 (94.5)	303 (93.8)	129 (96.3)	0.29
NDI§, n (%)				0.24
<25^th^ percentile (least deprived)	114 (25.0)	79 (24.6)	35 (26.1)	
25^th^ to <50^th^ percentile	115 (25.3)	89 (27.7)	26 (19.4)	
50^th^ to <75^th^ percentile	120 (26.4)	84 (26.2)	36 (26.9)	
75^th^ percentile or higher (most deprived)	106 (23.3)	69 (21.5)	37 (27.6)	
BMI§ (kg/m^2^), median (IQR)	28.0 (24.0–32.6)	28.0 (23.9–32.7)	28.2 (24.8–32.0)	0.83
Obese (≥30.0), n (%)	170 (37.5)	123 (38.1)	47 (36.2)	0.70
Unhoused status, n (%)	6 (1.3)	1 (0.3)	5 (3.7)	0.005
Alcohol use disorder, n (%)	39 (8.5)	24 (7.4)	15 (11.2)	0.28
Injection drug use, n (%)	22 (4.8)	13 (4.0)	9 (6.7)	0.21
Chronic steroid use, n (%)	32 (7.0)	29 (9.0)	3 (2.2)	0.01
Solid organ transplant, n (%)	6 (1.3)	6 (1.9)	0 (0.0)	0.19
HIV, n (%)	6 (1.3)	2 (0.6)	4 (3.0)	0.06
Diabetes mellitus, n (%)	158 (34.6)	116 (35.9)	42 (31.3)	0.35
Liver disease, n (%)	26 (5.7)	21 (6.5)	5 (3.7)	0.24
Kidney disease, n (%)	119 (26.0)	84 (26.0)	35 (26.1)	0.98
Recent infection, n (%)	192 (42.0)	144 (44.6)	48 (35.8)	0.14
Spine instrumentation, n (%)	49 (10.7)	34 (10.5)	15 (11.2)	0.70
Indwelling vascular catheter, n (%)	52 (11.4)	42 (13.0)	10 (7.5)	0.15

* Age and NDI were at study index date; body mass index was closest to the index date and within ±30 days of study index date; recent infection and indwelling vascular catheter were within 90 days prior to study index date; spine instrumentation was within 30 days prior to study index date; all other clinical characteristics were within one year prior to study index date.

† Comparisons were made using chi-squared or Fisher exact test for categorical variables and Wilcoxon-Mann-Whitney tests for nonparametric continuous variables.

‡ Other race/ethnicity: Multiple race (n=15); Native American (n=4); Unknown (n=3)

§ Missing values: Preferred language (n=25); NDI (n=2); BMI (n=4)

*SEA*, spinal epidural abscess; *IQR*, interquartile range; *NDI*, Neighborhood Deprivation Index; *BMI*, body mass index; *kg/m^2^*, kilogram per meter squared.

**Table 2:** Spinal epidural abscess patient characteristics at diagnosis.

Characteristics*	All SEA patients (N=457, 100%)	Potential delay (n=323, 70.7%)	No potential delay (n=134, 29.3%)	P-value†
Vital signs on index visit‡, n (%)				
Temperature, elevated (>100.4°F)	132 (29.3)	87 (27.3)	45 (34.4)	0.13
Respiration, high (>20/min)	169 (37.6)	117 (36.9)	52 (39.7)	0.58
Heart rate, high (>90/bpm)	321 (71.3)	223 (69.9)	98 (74.8)	0.30
Systolic, low (<90 mm Hg)	34 (7.6)	25 (7.8)	9 (6.9)	0.72
History and exam findings, n (%)				
Fever or chills	165 (36.1)	111 (34.4)	54 (40.3)	0.11
Radicular pain	165 (36.1)	125 (38.7)	40 (29.8)	0.19
Neck/back pain/spine tenderness	417 (91.2)	295 (91.3)	122 (91.0)	0.94
Sensory changes or symptoms	86 (18.8)	64 (19.8)	22 (16.4)	0.25
Weakness or motor findings	129 (28.2)	88 (27.2)	41 (30.6)	0.09
Back or neck pain, fever, and weakness	49 (10.7)	31 (9.6)	18 (13.4)	0.23
Cauda equina symptoms / findings (new bowel or bladder incontinence, decreased rectal tone, post void residual >200 mL)	57 (12.5)	44 (13.6)	13 (9.7)	0.15
Abscess location, n (%)				0.16
Cervical	50 (10.9)	40 (12.4)	10 (7.5)	
Thoracic	77 (16.8)	53 (16.4)	24 (17.9)	
Lumbar	235 (51.4)	170 (52.6)	65 (48.5)	
Multiple	95 (20.8)	60 (18.6)	35 (26.1)	
Laboratory data§				
WBC, median (IQR)	13.2 (8.9–16.9)	12.1 (8.9–16.4)	12.3 (9.0–17.7)	0.89
Neutrophil, median (IQR)	10.0 (6.5–12.9)	9.1 (6.5–12.6)	10.0 (6.3–14.1)	0.20
ESR (mm/hr), median (IQR)	77.1 (54.0–101.5)	84.5 (58.0–102.0)	78.0 (48.0–100.0)	0.28
CRP (mg/L), median (IQR)	15.3 (5.8–23.2)	14.0 (5.7–23.0)	12.7 (6.7–23.6)	0.46
Blood or abscess fluid culture, n (%)				0.46
*Staphylococcus aureus*, methicillin-sensitive	137 (31.0)	99 (31.7)	38 (29.2)	
*S. aureus*, methicillin-resistant	25 (5.7)	14 (4.5)	11 (8.5)	
*S. aureus*, coagulase-negative	9 (2.0)	8 (2.6)	1 (0.8)	
Other bacterial	90 (20.4)	64 (20.5)	26 (20.0)	
Other (Cryptosporidium, fungal)	2 (0.0)	1 (0.3)	1 (0.8)	
No growth	179 (42.4)	126 (40.4)	53 (40.8)	

* Vital sign measurements were within ±1 day of study index date; lab results were within ±3 days prior to study index date; all other clinical characteristics were within one year prior to study index date.

† Comparisons were made using chi-squared or Fisher’s exact tests for categorical variables and Wilcoxon-Mann-Whitney tests for nonparametric continuous variables.

‡ Missing vitals values: temperature (n=7); respiration (n=8); heart rate (n=7); blood pressure (n=7)

§ Missing lab values: WBC (n=18); neutrophil (n=73); ESR (n=168); CRP (n=166); blood/abscess cultures (n=15)

*SEA*, Spinal epidural abscess; *WBC*, white blood cell; *ESR*, erythrocyte sedimentation rate; *CRP*, C-reactive protein; *IQR*, interquartile range.

**Table 3:** Number of visits prior to diagnosis in cases of delayed diagnosis.

Number of visits	Frequency	Percent	Cumulative frequency	Cumulative percent
1	138	42.72	138	42.72
2	85	26.32	223	69.04
3	47	14.55	270	83.59
4	30	9.29	300	92.88
5	11	3.41	311	96.28
6	7	2.17	318	98.45
7	3	0.93	321	99.38
8	1	0.31	322	99.69
9	1	0.31	323	100.00
